# Supplementary material for: Modeling glioblastoma heterogeneity as a dynamic network of cell states
Source: Mol Syst Biol. 2021 Sep 16;17(9):e10105. doi: 10.15252/msb.202010105 (PMC8444284; doi:10.15252/msb.202010105)
Supplement: Supplementary file 5 — Source Data for Figure 3 [file MSB-17-e10105-s001.zip › Figure3A_sourcedata/GSEA_3065/nosigdb_state2.GseaPreranked.1622718419215/RICHARDS_ZHONG_NATURE_2018_NPCS_UPREG.html]

Details for gene set RICHARDS\_ZHONG\_NATURE\_2018\_NPCS\_UPREG[GSEA]

|  || Dataset | state2 |
| Phenotype | NoPhenotypeAvailable |
| Upregulated in class | na\_pos |
| GeneSet | RICHARDS\_ZHONG\_NATURE\_2018\_NPCS\_UPREG |
| Enrichment Score (ES) | 0.91303825 |
| Normalized Enrichment Score (NES) | 3.0887942 |
| Nominal p-value | 0.0 |
| FDR q-value | 0.0 |
| FWER p-Value | 0.0 |
Table: GSEA Results Summary

  

Fig 1: Enrichment plot: RICHARDS\_ZHONG\_NATURE\_2018\_NPCS\_UPREG      
 Profile of the Running ES Score & Positions of GeneSet Members on the Rank Ordered List

  

| PROBE | GENE SYMBOL | GENE\_TITLE | RANK IN GENE LIST | RANK METRIC SCORE | RUNNING ES | CORE ENRICHMENT || 1 | HIST1H4C |  |  | 0 | 1.309 | 0.0308 | Yes |
| 2 | HMGB2 |  |  | 4 | 0.823 | 0.0498 | Yes |
| 3 | CENPU |  |  | 6 | 0.763 | 0.0676 | Yes |
| 4 | H2AFZ |  |  | 8 | 0.735 | 0.0848 | Yes |
| 5 | UBE2C |  |  | 9 | 0.704 | 0.1014 | Yes |
| 6 | H2AFX |  |  | 10 | 0.675 | 0.1172 | Yes |
| 7 | DHFR |  |  | 11 | 0.644 | 0.1324 | Yes |
| 8 | UBE2T |  |  | 12 | 0.643 | 0.1475 | Yes |
| 9 | MCM7 |  |  | 13 | 0.626 | 0.1622 | Yes |
| 10 | TOP2A |  |  | 15 | 0.596 | 0.1761 | Yes |
| 11 | PCNA |  |  | 16 | 0.595 | 0.1901 | Yes |
| 12 | MKI67 |  |  | 17 | 0.590 | 0.2040 | Yes |
| 13 | CDK1 |  |  | 18 | 0.577 | 0.2175 | Yes |
| 14 | MAD2L1 |  |  | 20 | 0.563 | 0.2307 | Yes |
| 15 | ATAD2 |  |  | 21 | 0.563 | 0.2439 | Yes |
| 16 | RRM2 |  |  | 22 | 0.556 | 0.2570 | Yes |
| 17 | CKS1B |  |  | 23 | 0.554 | 0.2700 | Yes |
| 18 | DUT |  |  | 24 | 0.552 | 0.2830 | Yes |
| 19 | CLSPN |  |  | 27 | 0.536 | 0.2953 | Yes |
| 20 | CKS2 |  |  | 28 | 0.532 | 0.3079 | Yes |
| 21 | TPX2 |  |  | 29 | 0.531 | 0.3204 | Yes |
| 22 | CENPF |  |  | 34 | 0.518 | 0.3321 | Yes |
| 23 | HMGN2 |  |  | 35 | 0.512 | 0.3441 | Yes |
| 24 | RPA3 |  |  | 36 | 0.511 | 0.3562 | Yes |
| 25 | GINS2 |  |  | 37 | 0.506 | 0.3681 | Yes |
| 26 | SMC2 |  |  | 39 | 0.493 | 0.3795 | Yes |
| 27 | ORC6 |  |  | 40 | 0.493 | 0.3911 | Yes |
| 28 | SMC4 |  |  | 42 | 0.489 | 0.4025 | Yes |
| 29 | TMPO |  |  | 43 | 0.489 | 0.4140 | Yes |
| 30 | CENPK |  |  | 44 | 0.488 | 0.4255 | Yes |
| 31 | ZWINT |  |  | 45 | 0.488 | 0.4370 | Yes |
| 32 | PBK |  |  | 46 | 0.484 | 0.4483 | Yes |
| 33 | NUSAP1 |  |  | 47 | 0.483 | 0.4597 | Yes |
| 34 | KPNA2 |  |  | 50 | 0.472 | 0.4705 | Yes |
| 35 | MCM3 |  |  | 51 | 0.464 | 0.4815 | Yes |
| 36 | TUBA1B |  |  | 52 | 0.462 | 0.4923 | Yes |
| 37 | CENPM |  |  | 54 | 0.460 | 0.5030 | Yes |
| 38 | UHRF1 |  |  | 57 | 0.454 | 0.5135 | Yes |
| 39 | BIRC5 |  |  | 61 | 0.443 | 0.5236 | Yes |
| 40 | PTTG1 |  |  | 63 | 0.439 | 0.5338 | Yes |
| 41 | GMNN |  |  | 64 | 0.435 | 0.5440 | Yes |
| 42 | FBXO5 |  |  | 67 | 0.420 | 0.5537 | Yes |
| 43 | CCNB1 |  |  | 68 | 0.420 | 0.5635 | Yes |
| 44 | RRM1 |  |  | 72 | 0.412 | 0.5729 | Yes |
| 45 | MCM4 |  |  | 79 | 0.399 | 0.5816 | Yes |
| 46 | H2AFV |  |  | 82 | 0.397 | 0.5907 | Yes |
| 47 | MCM5 |  |  | 83 | 0.394 | 0.6000 | Yes |
| 48 | KIF15 |  |  | 86 | 0.392 | 0.6090 | Yes |
| 49 | CENPN |  |  | 87 | 0.390 | 0.6182 | Yes |
| 50 | GTSE1 |  |  | 89 | 0.389 | 0.6272 | Yes |
| 51 | DTYMK |  |  | 94 | 0.379 | 0.6357 | Yes |
| 52 | CDKN3 |  |  | 97 | 0.376 | 0.6443 | Yes |
| 53 | CDCA5 |  |  | 101 | 0.373 | 0.6527 | Yes |
| 54 | SPC25 |  |  | 104 | 0.372 | 0.6613 | Yes |
| 55 | FEN1 |  |  | 107 | 0.371 | 0.6698 | Yes |
| 56 | NDC80 |  |  | 115 | 0.354 | 0.6773 | Yes |
| 57 | KIF23 |  |  | 117 | 0.351 | 0.6855 | Yes |
| 58 | ASPM |  |  | 122 | 0.342 | 0.6931 | Yes |
| 59 | TUBB4B |  |  | 125 | 0.340 | 0.7009 | Yes |
| 60 | KIF11 |  |  | 129 | 0.335 | 0.7084 | Yes |
| 61 | TACC3 |  |  | 130 | 0.334 | 0.7163 | Yes |
| 62 | SNRPB |  |  | 132 | 0.332 | 0.7240 | Yes |
| 63 | MELK |  |  | 133 | 0.332 | 0.7318 | Yes |
| 64 | CCNA2 |  |  | 135 | 0.331 | 0.7394 | Yes |
| 65 | ANP32E |  |  | 136 | 0.330 | 0.7472 | Yes |
| 66 | MCM6 |  |  | 149 | 0.311 | 0.7532 | Yes |
| 67 | CKAP2 |  |  | 151 | 0.308 | 0.7604 | Yes |
| 68 | KIF22 |  |  | 154 | 0.306 | 0.7674 | Yes |
| 69 | CDCA3 |  |  | 160 | 0.304 | 0.7740 | Yes |
| 70 | HELLS |  |  | 161 | 0.304 | 0.7811 | Yes |
| 71 | AURKB |  |  | 163 | 0.303 | 0.7881 | Yes |
| 72 | BTG3 |  |  | 173 | 0.295 | 0.7941 | Yes |
| 73 | ITGB3BP |  |  | 177 | 0.294 | 0.8007 | Yes |
| 74 | CDCA4 |  |  | 184 | 0.292 | 0.8069 | Yes |
| 75 | PAICS |  |  | 187 | 0.291 | 0.8136 | Yes |
| 76 | NUF2 |  |  | 188 | 0.287 | 0.8203 | Yes |
| 77 | CCNB2 |  |  | 190 | 0.286 | 0.8269 | Yes |
| 78 | CDC20 |  |  | 193 | 0.284 | 0.8334 | Yes |
| 79 | HMGB3 |  |  | 200 | 0.282 | 0.8394 | Yes |
| 80 | HJURP |  |  | 209 | 0.277 | 0.8451 | Yes |
| 81 | C19orf48 |  |  | 215 | 0.274 | 0.8510 | Yes |
| 82 | MCM2 |  |  | 228 | 0.262 | 0.8558 | Yes |
| 83 | WDR34 |  |  | 235 | 0.260 | 0.8613 | Yes |
| 84 | KIF2C |  |  | 252 | 0.251 | 0.8655 | Yes |
| 85 | MIS18BP1 |  |  | 257 | 0.246 | 0.8709 | Yes |
| 86 | PHGDH |  |  | 271 | 0.243 | 0.8752 | Yes |
| 87 | PRR11 |  |  | 285 | 0.238 | 0.8794 | Yes |
| 88 | BUB3 |  |  | 311 | 0.228 | 0.8821 | Yes |
| 89 | CKAP2L |  |  | 327 | 0.224 | 0.8857 | Yes |
| 90 | BUB1 |  |  | 335 | 0.222 | 0.8902 | Yes |
| 91 | DLGAP5 |  |  | 371 | 0.211 | 0.8914 | Yes |
| 92 | CENPW |  |  | 373 | 0.211 | 0.8962 | Yes |
| 93 | HAT1 |  |  | 381 | 0.209 | 0.9004 | Yes |
| 94 | GNG5 |  |  | 389 | 0.207 | 0.9045 | Yes |
| 95 | SPAG5 |  |  | 439 | 0.192 | 0.9037 | Yes |
| 96 | KNSTRN |  |  | 483 | 0.183 | 0.9034 | Yes |
| 97 | DEPDC1B |  |  | 505 | 0.179 | 0.9054 | Yes |
| 98 | LDHA |  |  | 506 | 0.179 | 0.9096 | Yes |
| 99 | PRC1 |  |  | 514 | 0.178 | 0.9130 | Yes |
| 100 | ID4 |  |  | 705 | 0.150 | 0.8962 | No |
| 101 | TGIF1 |  |  | 1150 | 0.108 | 0.8510 | No |
| 102 | CLIC1 |  |  | 1291 | 0.099 | 0.8383 | No |
| 103 | UCP2 |  |  | 1329 | 0.096 | 0.8366 | No |
| 104 | CNTLN |  |  | 1386 | 0.093 | 0.8328 | No |
| 105 | SOX9 |  |  | 1466 | 0.088 | 0.8264 | No |
| 106 | RNASEH2A |  |  | 1579 | 0.082 | 0.8163 | No |
| 107 | CDCA7 |  |  | 1684 | 0.078 | 0.8070 | No |
| 108 | TMEM98 |  |  | 1991 | 0.065 | 0.7756 | No |
| 109 | OIP5 |  |  | 2175 | 0.058 | 0.7574 | No |
| 110 | GLI3 |  |  | 3012 | 0.034 | 0.6684 | No |
| 111 | PAX6 |  |  | 3226 | 0.029 | 0.6462 | No |
| 112 | HSPB1 |  |  | 3629 | 0.022 | 0.6036 | No |
| 113 | C21orf58 |  |  | 3849 | 0.018 | 0.5805 | No |
| 114 | PLIN2 |  |  | 4837 | 0.002 | 0.4746 | No |
| 115 | TAGLN2 |  |  | 5102 | -0.002 | 0.4463 | No |
| 116 | EFNB1 |  |  | 5978 | -0.016 | 0.3527 | No |
| 117 | HES1 |  |  | 6191 | -0.020 | 0.3304 | No |
| 118 | MDK |  |  | 6783 | -0.031 | 0.2677 | No |
| 119 | RPS27L |  |  | 7139 | -0.040 | 0.2306 | No |
| 120 | SFRP2 |  |  | 7843 | -0.061 | 0.1565 | No |
| 121 | SFRP1 |  |  | 8103 | -0.071 | 0.1304 | No |
| 122 | CREB5 |  |  | 8279 | -0.082 | 0.1135 | No |
| 123 | ZFHX4 |  |  | 8390 | -0.088 | 0.1038 | No |
| 124 | VIM |  |  | 8691 | -0.110 | 0.0742 | No |
| 125 | NOTCH1 |  |  | 8763 | -0.118 | 0.0693 | No |
| 126 | RCN1 |  |  | 8929 | -0.138 | 0.0549 | No |
Table: GSEA details [plain text format]

  

Fig 2: RICHARDS\_ZHONG\_NATURE\_2018\_NPCS\_UPREG: Random ES distribution      
 Gene set null distribution of ES for **RICHARDS\_ZHONG\_NATURE\_2018\_NPCS\_UPREG**
